# Publisher Correction: Visuo-frontal interactions during social learning in freely moving macaques

**DOI:** 10.1038/s41586-024-07301-7

**Published:** 2024-03-22

**Authors:** Melissa Franch, Sudha Yellapantula, Arun Parajuli, Natasha Kharas, Anthony Wright, Behnaam Aazhang, Valentin Dragoi

**Affiliations:** 1 Deparment of Neurobiology and Anatomy, McGovern Medical School, University of Texas, Houston, TX USA; 2 Department of Electrical and Computer Engineering, Rice University, Houston, TX USA; 3 Present Address: Neuroengineering Initiative, Rice University, Houston, TX USA; 4 Present Address: Houston Methodist Research Institute, Houston, TX USA

**Keywords:** Cooperation, Neural circuits, Extrastriate cortex, Social behaviour

Correction to: *Nature* 10.1038/s41586-024-07084-x Published online 14 February 2024

This paper was originally published under a standard Springer Nature license (© The Author(s), under a exclusive licence to Springer Nature Limited). It is now available as an open-access paper under a Creative Commons Attribution 4.0 International license, © The Author(s). In addition, the first names of all authors were originally published as initials only, and are now listed as full names. These errors have been corrected in the HTML and PDF versions of the article.

