# Supplementary material for: Visuo-frontal interactions during social learning in freely moving macaques
Source: Nature. 2024 Feb 14;627(8002):174–81. doi: 10.1038/s41586-024-07084-x (PMC10959748; doi:10.1038/s41586-024-07084-x)
Supplement: Supplementary file 1 — Reporting Summary [file 41586_2024_7084_MOESM1_ESM.pdf]

Corresponding author(s): Valentin Dragoi
Last updated by author(s): Dec 14, 2023

Reporting Summary

Nature Portfolio wishes to improve the reproducibility of the work that we publish. This form provides structure for consistency and transparency in reporting. For further information on Nature Portfolio policies, see our Editorial Policies and the Editorial Policy Checklist.

Statistics

For all statistical analyses, confirm that the following items are present in the figure legend, table legend, main text, or Methods section.

n/a	Confirmed	
☐	☒	The exact sample size (*n*) for each experimental group/condition, given as a discrete number and unit of measurement
☐	☒	A statement on whether measurements were taken from distinct samples or whether the same sample was measured repeatedly
☐	☒	The statistical test(s) used AND whether they are one- or two-sided<br>*Only common tests should be described solely by name; describe more complex techniques in the Methods section.*
☐	☒	A description of all covariates tested
☐	☒	A description of any assumptions or corrections, such as tests of normality and adjustment for multiple comparisons
☐	☒	A full description of the statistical parameters including central tendency (e.g. means) or other basic estimates (e.g. regression coefficient) AND variation (e.g. standard deviation) or associated estimates of uncertainty (e.g. confidence intervals)
☐	☒	For null hypothesis testing, the test statistic (e.g. *F*, *t*, *r*) with confidence intervals, effect sizes, degrees of freedom and *P* value noted<br>*Give P values as exact values whenever suitable.*
☒	☐	For Bayesian analysis, information on the choice of priors and Markov chain Monte Carlo settings
☒	☐	For hierarchical and complex designs, identification of the appropriate level for tests and full reporting of outcomes
☐	☒	Estimates of effect sizes (e.g. Cohen's *d*, Pearson's *r*), indicating how they were calculated

Our web collection on statistics for biologists contains articles on many of the points above.

Software and code

Policy information about availability of computer code

Data collection	The neural data was collected using the the 'central' software (BlackRock Microsystems). The behavioral data and video data was collected using custom code in Matlab 2020b. The eye position and pupil size data was collected using the hardware and software provided by ISCAN Inc.
Data analysis	The neural data was pre-processed using Offline Sorter v4 (Plexon inc.). The behavioral, neural and part of the video material was processed using costum code in Matlab 2020b. The rest of the video material was processed using DeepLabCut (open source; version 2.0).

For manuscripts utilizing custom algorithms or software that are central to the research but not yet described in published literature, software must be made available to editors and reviewers. We strongly encourage code deposition in a community repository (e.g. GitHub). See the Nature Portfolio guidelines for submitting code & software for further information.

Data

Policy information about availability of data

All manuscripts must include a data availability statement. This statement should provide the following information, where applicable:

- Accession codes, unique identifiers, or web links for publicly available datasets
- A description of any restrictions on data availability
- For clinical datasets or third party data, please ensure that the statement adheres to our policy

All source data used to generate experimental Figures is available at https://zenodo.org/records/10384447. The raw data that support the findings of this study are available from the corresponding authors upon reasonable request.

Research involving human participants, their data, or biological material

Policy information about studies with human participants or human data. See also policy information about sex, gender (identity/presentation), and sexual orientation and race, ethnicity and racism.

Reporting on sex and gender	NA
Reporting on race, ethnicity, or other socially relevant groupings	NA
Population characteristics	NA
Recruitment	NA
Ethics oversight	NA

Note that full information on the approval of the study protocol must also be provided in the manuscript.

Field-specific reporting

Please select the one below that is the best fit for your research. If you are not sure, read the appropriate sections before making your selection.

☒ Life sciences ☐ Behavioural & social sciences ☐ Ecological, evolutionary & environmental sciences

For a reference copy of the document with all sections, see nature.com/documents/nr-reporting-summary-flat.pdf

Life sciences study design

All studies must disclose on these points even when the disclosure is negative.

Sample size	We conducted this cooperation study with two distinct monkey pairs consisting of three monkeys, but only recorded neural and eye tracking data from two monkeys in order to achieve robustness for the behavioral and electrophysiological results despite possible differences in the behavioral strategies between monkeys (all results were consistent across animals). We limited the number of neurally recorded monkeys to two for the main results to meet the requirements of lab animal use regulations that requires minimizing the number of animals in each study. Choosing the sample size of two is typical in electrophisiological studies in monkeys.
Data exclusions	For SVM modeling and cross-correlation, some sessions were excluded if they did not contain enough observations to perform the analysis.
Replication	Results were replicated across sessions and animals. The main experiments were repeated up to 20 times in each animal, and control experiments up to 10 times in each animal.
Randomization	Two distinct pairs of familiar animals completed the same cooperation experiment at separate times, with data always recorded from the subordinate animal in the pair. In control experiments, conditions were interleaved.
Blinding	Since no group allocation was done in this study, blinding was not required.

Reporting for specific materials, systems and methods

We require information from authors about some types of materials, experimental systems and methods used in many studies. Here, indicate whether each material, system or method listed is relevant to your study. If you are not sure if a list item applies to your research, read the appropriate section before selecting a response.

Materials & experimental systems

n/a	Involved in the study
☒	☐ Antibodies
☒	☐ Eukaryotic cell lines
☒	☐ Palaeontology and archaeology
☐	☒ Animals and other organisms
☒	☐ Clinical data
☒	☐ Dual use research of concern
☒	☐ Plants

Methods

n/a	Involved in the study
☒	☐ ChIP-seq
☒	☐ Flow cytometry
☒	☐ MRI-based neuroimaging

Animals and other research organisms

Policy information about studies involving animals; ARRIVE guidelines recommended for reporting animal research, and Sex and Gender in Research

Laboratory animals	Three adult male rhesus monkeys (Macaca mulatta) were used in this study, including Monkey M age 11, Monkey R ages 10 and 12, and Monkey G age 16 during the time of experiments.
Wild animals	The study did not involve wild animals.
Reporting on sex	Due to the limited number of animals used for the main study (n=3), no reporting on sex or gender was possible.
Field-collected samples	The study did not include field-collected samples
Ethics oversight	All experiments were performed in accordance with The Animal Welfare Committee (AWC) and the Institutional Animal Care and Use Committee (IACUC) for McGovern Medical School at The University of Texas Health Science Center at Houston.

Note that full information on the approval of the study protocol must also be provided in the manuscript.

Plants

Seed stocks	*Report on the source of all seed stocks or other plant material used. If applicable, state the seed stock centre and catalogue number. If plant specimens were collected from the field, describe the collection location, date and sampling procedures.*
Novel plant genotypes	*Describe the methods by which all novel plant genotypes were produced. This includes those generated by transgenic approaches, gene editing, chemical/radiation-based mutagenesis and hybridization. For transgenic lines, describe the transformation method, the number of independent lines analyzed and the generation upon which experiments were performed. For gene-edited lines, describe the editor used, the endogenous sequence targeted for editing, the targeting guide RNA sequence (if applicable) and how the editor was applied.*
Authentication	*Describe any authentication procedures for each seed stock used or novel genotype generated. Describe any experiments used to assess the effect of a mutation and, where applicable, how potential secondary effects (e.g. second site T-DNA insertions, mosiacism, off-target gene editing) were examined.*
